# FKBP51 employs both scaffold and isomerase functions to promote NF-κB activation in melanoma

**DOI:** 10.1093/nar/gkv615

**Published:** 2015-06-22

**Authors:** Simona Romano, Yichuan Xiao, Mako Nakaya, Anna D'Angelillo, Mikyoung Chang, Jin Jin, Felix Hausch, Mariorosario Masullo, Xixi Feng, Maria Fiammetta Romano, Shao-Cong Sun

**Affiliations:** 1 Department of Molecular Medicine and Medical Biotechnologies, Federico II University, Naples 80131, Italy; 2 Department of Immunology, The University of Texas MD Anderson Cancer Center, Houston, TX 77030, USA; 3 Institute of Health Sciences, Shanghai Institutes for Biological Sciences, Chinese Academy of Sciences/Shanghai Jiao Tong University School of Medicine, Shanghai 200031, China; 4 Department Translational Research in Psychiatry, Max Planck Institute of Psychiatry, München 80804, Germany; 5 Department of Movement Sciences and Wellness, University of Naples ‘Parthenope’, Naples 80133, Italy

## Abstract

Melanoma is the most aggressive skin cancer; its prognosis, particularly in advanced stages, is disappointing largely due to the resistance to conventional anticancer treatments and high metastatic potential. NF-κB constitutive activation is a major factor for the apoptosis resistance of melanoma. Several studies suggest a role for the immunophilin FKBP51 in NF-κB activation, but the underlying mechanism is still unknown. In the present study, we demonstrate that FKBP51 physically interacts with IKK subunits, and facilitates IKK complex assembly. FKBP51-knockdown inhibits the binding of IKKγ to the IKK catalytic subunits, IKK-α and -β, and attenuates the IKK catalytic activity. Using FK506, an inhibitor of the FKBP51 isomerase activity, we found that the IKK-regulatory role of FKBP51 involves both its scaffold function and its isomerase activity. Moreover, FKBP51 also interacts with TRAF2, an upstream mediator of IKK activation. Interestingly, both FKBP51 TPR and PPIase domains are required for its interaction with TRAF2 and IKKγ, whereas only the TPR domain is involved in interactions with IKKα and β. Collectively, these results suggest that FKBP51 promotes NF-κB activation by serving as an IKK scaffold as well as an isomerase. Our findings have profound implications for designing novel melanoma therapies based on modulation of FKBP51.

## INTRODUCTION

FK506 binding proteins (FKBPs) are multifunctional proteins highly conserved across the species and abundantly expressed in the cell. FKBPs belong to the family of immunophilins, which includes also cyclophilins (Cyp) (1). The term immunophilin derives from the immunosuppressant action of the complexes formed between these proteins and the natural products cyclosporine A, FK506 and rapamycin (1). In addition to a well-established role in immunosuppression, immunophilins modulate several signal transduction pathways in the cell, due to their isomerase activity and the capability to interact with other proteins, inducing changes in conformation and function of partner proteins (2,3). Many immunophilins have a peptidyl-prolyl *cis*–*trans* isomerase activity (PPIase), which is inhibited by drug ligand binding (1). FKBP51, encoded by the *FKBP5* gene, has a C-terminal tetratricopeptide repeat (TPR) domain, known to be involved in interaction with Hsp90 as well as other proteins (4,5). This structural feature suggests that FKBP51 may share some functions with heat shock proteins (6). The N-terminal region of FKBP51 contains two FKBP-like domains (FK1 and FK2). Only the FK1 domain is capable of PPIase activity and immunosuppressant drug binding, while the FK2 domain seems to have a structural role (5,6).

FKBP51 is highly expressed in melanoma (7,8) and plays an important role in tumor progression and metastasis (9,10). Several lines of evidence support an essential role for FKBP51 in the control of NF-κB activation (7,8,11–14). An interaction of FKBP51 with IKKα was firstly identified in a study mapping the protein interaction network of the TNFα/NF-κB pathway (13). In addition, tandem affinity purification-tagged FKBP51 resulted in the identification of several other kinases, indicating that FKBP51 might be a prominent multifunctional kinase cofactor (15). RNA interference for FKBP51 confirmed an essential role for this immunophilin in the overall signaling process of NF-κB activation (13). Other studies identified FKBP51 as an essential factor for chemotherapy induced NF-κB activation in melanoma and leukemia (7,11). Rapamycin, indeed, counteracted NF-κB activation induced by doxorubicin and decreased the nuclear translocation of NF-κB by inhibiting the ability of IKK kinase complex to phosphorylate IκB. The effect of rapamycin was reproduced by FKBP51 depletion (7), while the over expression of p65/RelA counteracted the action of the macrocyclic agent (10). This immunophilin was also found essential in the activation of NF-κB by ionizing radiation. Irradiated FKBP51-silenced melanoma cells showed reduced clonogenic potential due to impaired capability to activate NF-κB. Evidence of FKBP51 involvement in radioresistance was also provided by studies with a melanoma xenograft mouse model (8). In addition to melanoma and leukemia, the relevant roles of FKBP51 in NF-κB activation, chemoresistance, and tumor growth have also been demonstrated in glioma (14). In ovarian cancer, FKBP51 was recently identified as critical factor of chemoresistance (16).

Despite the numerous studies in support of a role for FKBP51 in promoting NF-κB activation, and IKK complex function (7,8,11–14), molecular mechanisms underlying the interplay between this large immunophilin and -κB signal transduction proteins are still unknown. The present study supports the conclusion that, both enzymatic and scaffold functions of FKBP51, are essential for IKK complex assembly and activation. In addition, we show that FKBP51 is essential for IKKγ recruitment to TRAF2, an adaptor protein central in the IKK kinase activation cascade, that links IKK to TAK1.

## MATERIALS AND METHODS

### Cell cultures, plasmids, antibodies and reagents

The melanoma cell lines SAN and A375 were cultured as described (8). The pcDNA expression vectors encoding hemagglutinin (HA)-tagged human IKKα, IKKβ, IKKγ, TAK1 and TRAF2 have been described previously (17–20); the pCMV Myc-DDK-tagged human FKBP51-transcript variant 1 (canonical FKBP51 [Myc-Flag-FKBP51]) and FKBP51-transcript variant 4 (short FKBP51 lacking of TPR domains [Myc-Flag-FKBP51s]) were from OriGene Technologies (MD, USA); samples of plasmids encoding pRK5 Flag-tagged FKBP51 or mutants (pRK5 empty vector [EV]; pRK5 FKBP51 WT [Flag-FKBP51]; pRK5 FKBP51-TPR-mut [Flag-FKBP51-mutTPR; K352A/R356A]; pRK5 FKBP51-PPIase-mut [Flag-FKBP51-mutPPIase; FD67DV]) were kindly provided by Theo Rein (Max Planck Institute of Psychiatry, Munich, Germany) (21). Antibodies to human Bax (B-9, mouse monoclonal), anti-IKKγ (FL419, rabbit polyclonal), anti-IKKα/β (H-470, rabbit polyclonal), anti-IKKβ (H4, mouse monoclonal), anti-IκBα (C-21, rabbit polyclonal), anti-lamin B (C-20, goat polyclonal), anti-TRAF2 (C-20, rabbit polyclonal), Hsp60 (H300, rabbit polyclonal), anti-Tak1 (M-579, rabbit polyclonal) were from Santa Cruz Biotechnology (CA, USA). Anti-FKBP51 was from Novus biological (CO, USA). Anti-β Actin (C-4, mouse monoclonal), anti-γ-Tubulin (GTU-88, mouse monoclonal), horseradish peroxidase-conjugated anti-hemagglutinin (HA-7) and anti-Flag (M2) were from Sigma Aldrich (MO, USA). Fluorescence-labeled antibody reagents are described below (see ‘Flow cytometry’ section). Human recombinant tumor necrosis factor α (TNFα), doxorubicin hydrochloride, etoposide, cyclosporin A and tacrolimus (FK506) were from Sigma-Aldrich.

### Knockdown of FKBP51 in melanoma cells

For the production of lentiviral particles, the lentiviral vector pGIPZ, either empty (pGIPZ) or encoding three different FKBP51-specific short hairpin RNA (pGIPZ-shFKBP51), was transfected into human embryonic kidney (HEK293) cells (by the calcium method) along with the packaging vectors psPAX2 and pMD2 (provided by X. Qin). Melanoma A375 and SAN cells were infected with lentivirus carrying either pGIPZ or pGIPZ-shFKBP51. After 24 h after the infection, cells were selected with 200 ng/ml puromycin (Sigma-Aldrich) and after further 48 h, cultures were enriched for infected cells by cell sorting by flow cytometry (based on GFP expression). Sequences of the three different pGIPZ-shFKBP51 are reported:

- shFKBP51.1_Mature Antisense: TTGTCTCCAATCATCGGCG
- shFKBP51.2_Mature Antisense: TATATAAGCTCAGCATTAG
- shFKBP51.3_Mature Antisense: TTCCAATTGGAATGTCGTG

### Coimmunoprecipitation, immunoblot, electrophoretic mobility-shift and kinase assays

Melanoma A375 and SAN cell lines were stimulated with 20 ng/ml TNFα. Total and subcellular extracts were prepared from the cells and subjected to coimmunoprecipitation (co-IP), immunoblot (IB) and electrophoretic mobility-shift assay (EMSA) as described (8,20). For co-IP, cell were lysed in RIPA buffer (50 mM Tris–HCl, pH7.4; 150 mM NaCl; 1% NP-40; 0.5% Na-deoxycholate; 1 mM ethylenediaminetetraacetic acid (EDTA); 1 mM phenylmethanesulfonylfluoride (PMSF); 1 mM dithiothreitol (DTT); protease inhibitor cocktail). PMSF and protease inhibitor cocktail were not added to the wash buffer. Antibodies used for co-IP: anti-FKBP51 antibodies were from Novus Biologicals (rabbit polyclonal, NB100-68240) and from Abnova (mouse polyclonal H00002289-B01P, Taipei, Taiwan); anti-IKKα antibodies were from Santa Cruz (rabbit polyclonal anti-IKKα, H-744; rabbit polyclonal anti-IKKα/β, H-470).

For protein-phosphorylation analysis and kinase assays (KA), cells were lysed in a kinase cell lysis buffer supplemented with phosphatase inhibitors. IKK was isolated by immunoprecipitation with anti-IKKγ (FL-419; Santa Cruz. Biote A moderate effect of FK506 on IKK activation was also detected in the FKBP51-knockdown cells, probably due to the incomplete depletion of FKBP51 in these cells chnology) and subjected to KA with a glutathione S-transferase fusion protein containing the amino-terminal 54 amino acids of IκBα (GST-IκBα [1-54]) as the substrate (20).

### qPCR

Total RNA was isolated from cells using Trizol (Invitrogen, Carlsbad, CA, USA) and 1 μg of each RNA was used for cDNA synthesis with iScript Reverse Transcriptase (Bio-Rad, Hercules, CA, USA). Gene expression was quantified by quantitative (q) PCR using SsoAdvanced Universal qPCR Supermixe (Bio-Rad) and specific qPCR primers for the relative quantitation of the transcripts, performed using co-amplified ribosomal β-Actin as an internal control for normalization. Oligo sequences are reported:

- hβ-Actin-Fw: 5′-CGAGGCCCAGAGCAAGAGAG-3′
- hβ-Actin-Rev: 5′-CGGTTGGCCTTAGGGTTCAG-3′
- hIκBα-Fw: 5′-CCAGGGCTATTCTCCCTACC-3′
- hIκBα-Rev: 5′-GCTCGTCCTCTGTGAACTCC-3′
- hBAX-Fw: 5′-GGACGAACTGGACAGTAACATG-3′
- hBAX-Rev: 5′-GTTGTCGCCCTTTTCTACTTTGC-3′
- hTRAF2-Fw: 5′-CAGTTCGGCCTTCCCAGATAA -3′
- hTRAF2-Rev: 5′-TCGTGGCAGCTCTCGTATTCTT-3′
- hCCND1-Fw: 5′-ACAAACAGATCATCCGCAAACAC-3′
- hCCND1-Rev: 5′-TGTTGGGGCTCCTCAGGTTC-3′

Validated FKBP1A qPCR primers were purchased from Qiagen (Philadelphia, PA, USA; QT00070742: Hs_FKBP1A_1_SG QuantiTect Primer Assay).

### Analysis of apoptosis

Apoptosis was measured using propidium iodide in double staining with annexin V-FITC (BD Biosciences, NJ, USA). Annexin-V binds to phosphatidylserine exposed on the outer leaflet of plasmamembrane of dying cells, Propidium iodide does not stain cells with intact membrane. Both early apoptosis (cell positive for annexin-V and negative for PI) and late apoptosis (cell double positive for annexin-V and PI) were measured with a flow cytometer (LSR II, BD Biosciences).

## RESULTS

### FKBP51 improves IKK enzymatic activity

**Figure 1.**
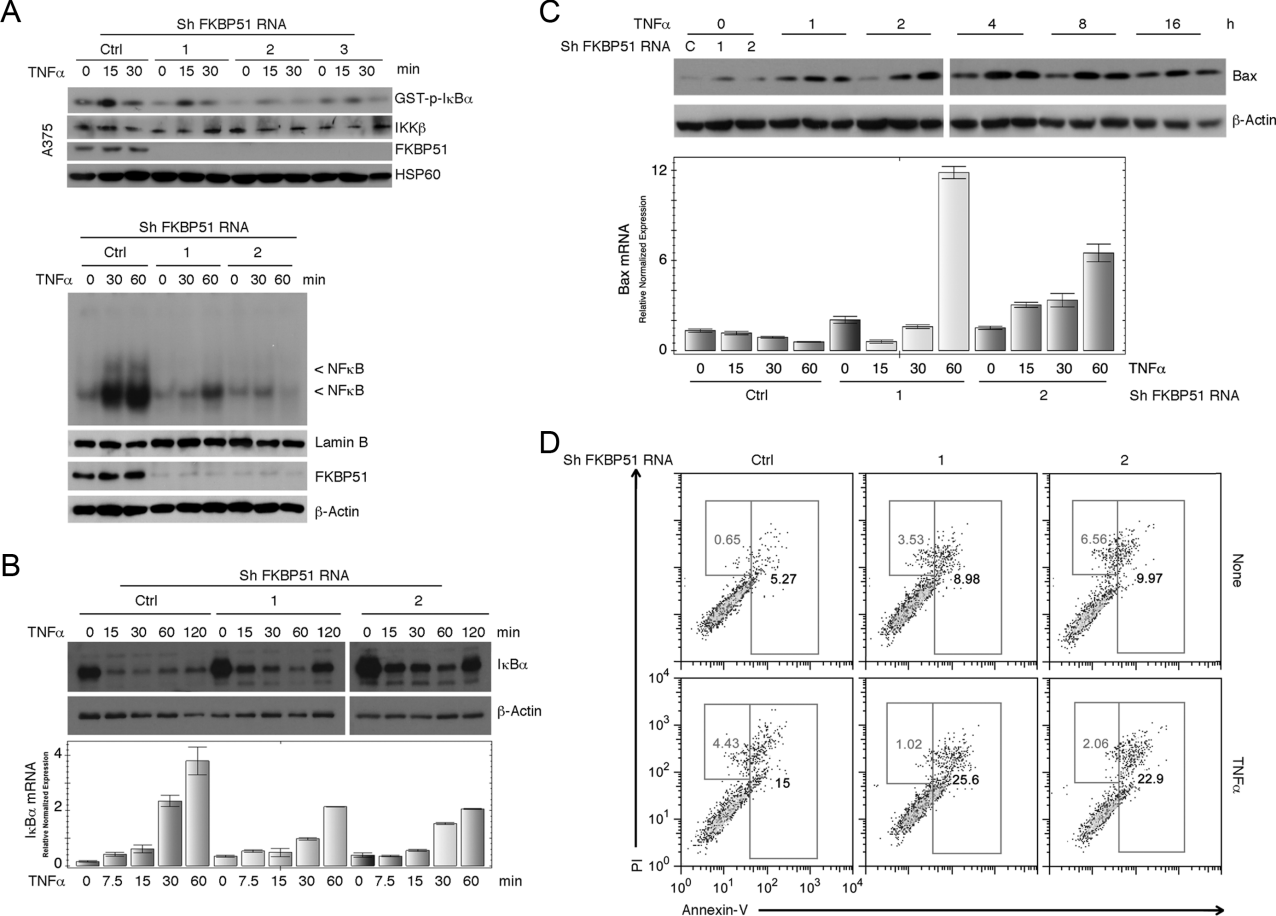
Effect of FKBP51 knockdown on NF-κB activation and apoptosis sensitivity in A375 melanoma cells. (**A**, upper) IB assay of IκBα phosphorylation levels in KA samples. IKK complexes were immunoprecipitated from melanoma cells stably knocked down with three different FKBP51 shRNAs (1, 2 and 3) or with a control shRNA (Ctrl) and stimulated with TNFα for 15 and 30 min. GST-IκBα served as substrate (top panel) to measure IKK activity. IB assay also monitored the IKKβ levels in immunoprecipitated-protein. Whole lysates showed the efficacy of FKBP51 knock down. HSP60 was used as a loading control. The reduced level of GST-p-IκBα in condition of FKBP51 knock down is consistent with a reduced phosphorylating capacity of immunoprecipitated IKK. (**A**, lower) EMSA of nuclear extracts of FKBP51-knocked down A375 cells, stimulated with TNFα for 30 and 60’. The band was generated by NF-κB binding to a ^32^P-radiolabeled probe. The bands indicated by the arrow are reduced in FKBP51 knock down cells. Nuclear extract was normalized using lamin B as a loading control. The expression of FKBP51 and β-actin in total lysates is also shown. (**B**) Assay of TNFα-induced changes in expression levels of IκBα protein (IB, upper) and mRNA (qPCR, lower). (**C** and **D**) Effect of FKBP51 knock down on TNFα-induced apoptosis. (**C**) Expression levels (protein and mRNA) of pro-apoptotic Bax. (**D**) Representative flow cytometric histograms of annex-V/PI staining. Data are representative of three independent experiments.

Previous studies have shown the pivotal role of FKBP51 in promoting activation of NF-κB in melanoma, induced by chemotherapeutics (7,11,14) or ionizing radiation (8). Since FKBP51 has been implicated as a cofactor of IKK complex (13), we examined the role of FKBP51 in maintaining IKK kinase activity by stably knocking down FKBP51 in melanoma cell lines. FKBP51 knockdown in A375 melanoma cells, using three different shRNAs, profoundly reduced the kinase activity of IKK after stimulation with TNFα (Figure 1a, upper). Consistent with this finding, EMSA showed a remarkable decrease of active NF-κB complexes (indicated by the arrow), in the nuclear extracts of TNFα-stimulated FKBP51-knockdown melanoma cells (Figure 1a, lower). Moreover, the TNFα-induced IκBα degradation, a central step in NF-κB activation, was attenuated in the FKBP51-knockdown cells (Figure 1b, upper). Since the gene encoding IκBα is the first transcriptional target of activated NF-κB, we also examined the level of IκBα mRNA. The induction of IκBα mRNA by TNFα was substantially inhibited in the FKBP51-silenced A375 cells (Figure 1b, lower), thus further confirming the reduced activation of NF-κB in these cells. It is worth noting that, in control cells, IκB-α production, which occurs at transcriptional level, apparently is not evident at protein level. This is consistent with high melanoma content in FKBP51, supporting an increased IKK activity and consequent IκB-α degradation. FKBP51 has been shown to inhibit apoptosis in melanoma cells (10–12). In agreement with these previous reports, we found that FKBP51 knockdown promoted TNFα-stimulated expression of the proapoptotic factor Bax, at both protein (Figure 1c, upper) and mRNA (Figure 1c, lower) levels. Accordingly, TNFα induced cell death to a greater extent in the FKBP51-silenced melanoma cells, compared to non-silenced control melanoma cells (Figure 1d). The FKBP51 knockdown also sensitized A375 cells to apoptosis induction by doxorubicin and etoposide (see supplementary information, Supplementary Figure S1). The effect of FKBP51 knockdown on IKK activity was not limited to A375 cells, since similar results were obtained with another melanoma cell line, SAN (see supplementary information, Supplementary Figure S2). Similarly to A375 cells, SAN melanoma cells depleted of FKBP51 showed impaired IKK kinase activity following TNFα stimulation, and a reduced NF-κB activation, in comparison with SAN control cells (Supplementary Figure S2). Interestingly, the FKBP51 knockdown attenuated the expression of Bcl-2 and concomitantly increased the expression of p53 in TNFα-stimulated SAN melanoma cells (Supplementary Figure S2).

### Both scaffold and isomerase functions of FKBP51 are essential for IKK activity

**Figure 2.**
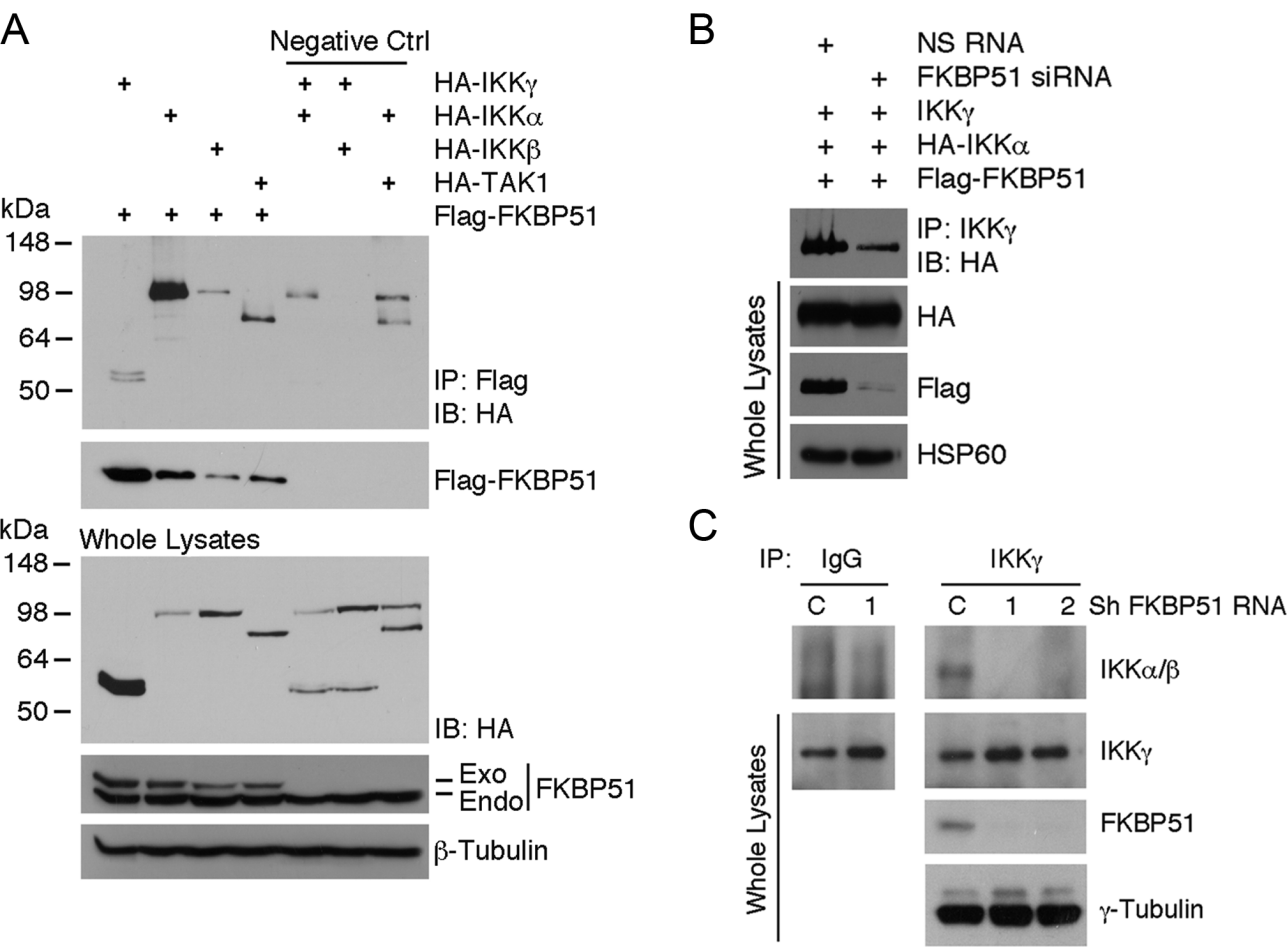
FKBP51 interacts with all IKK complex subunits and supports IKK assembly. (**A**) Immunoassay of HEK293 cells transfected with several combinations of expression vectors, namely HA-IKKγ, -IKKα, -IKKβ, -TAK1 and Flag-FKBP51. Anti-Flag immunoprecipitated proteins were IB assayed with anti-HA and anti-Flag (upper). IB analysis of total lysates is also shown (lower). (**B** and **C**) FKBP51 knock down impairs the IKKγ/IKKα/IKKβ interaction. (**B**) Immunoassay of HEK293 cells, silenced or not for FKBP51, and transfected with the expression vectors for IKKγ, HA-IKKα and Flag-FKBP51. IKKγ was immunoprecipitated from cell lysates; IP was analyzed by IB with anti-HA. (**C**) Endogenous IKKγ was immunoprecipitated from whole lysates of stably knocked down A375 melanoma cells (1,2) and the relative negetive control (C). Immunoprecipitated proteins were then assayed by IB. Data are representative of three independent experiments.

**Figure 3.**
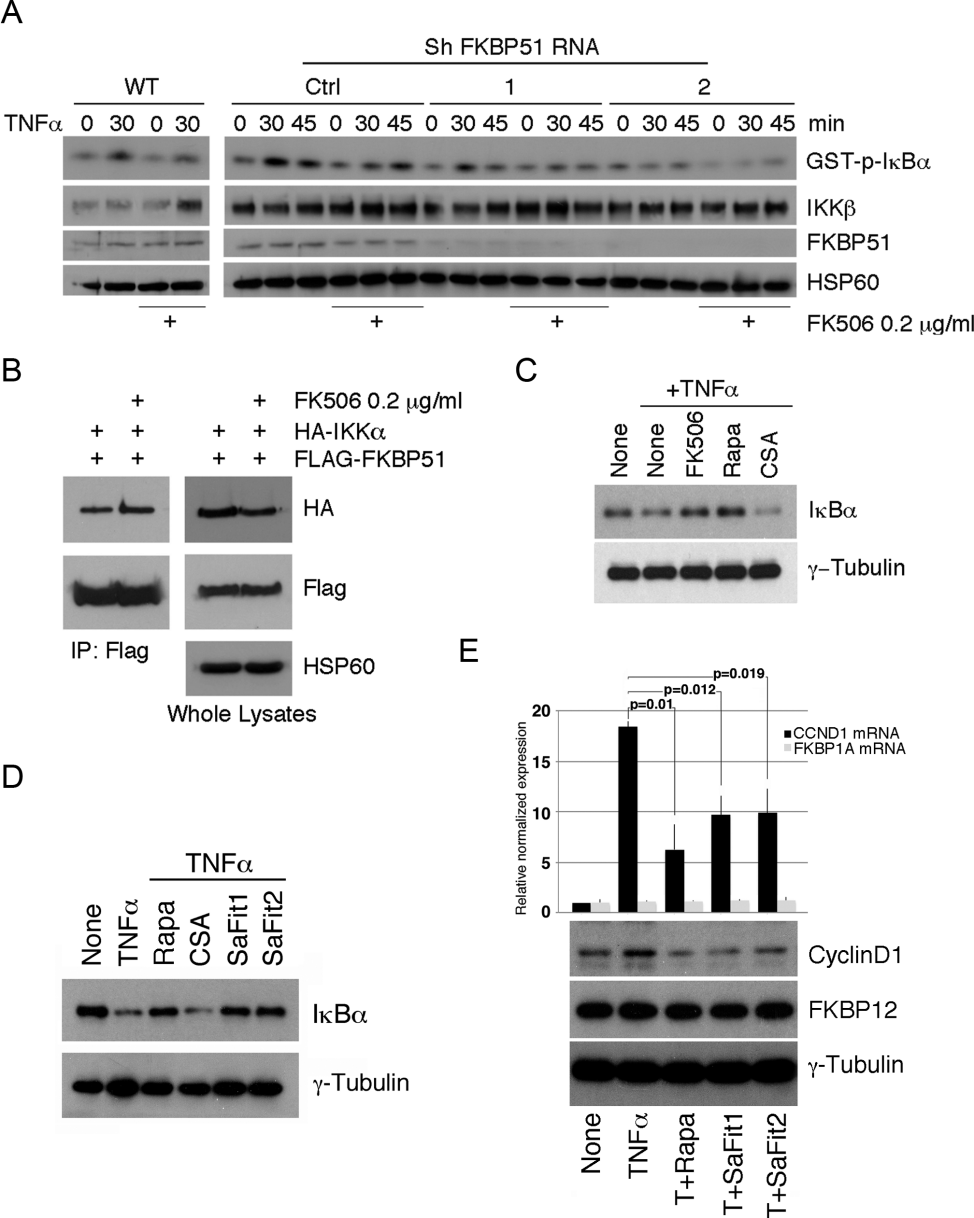
FKBP51 isomerase activity affects the enzymatic function of IKK kinase complex. (**A**) IB of IκBα phosphorylation levels of samples assayed in KA. IKK complexes were immunoprecipitated from WT and FKBP51-knocked down A375 cells, stimulated with TNFα for 30 and 45 min, in the presence or not of FK506. IKK activation was measured using GST-IκBα as substrate. IB also monitored the expression of IKKβ in immunoprecipitated protein and FKBP51 in total lysates. HSP60 served as loading control for total lysates. Data are representative of three independent experiments. (**B**) Immunoassay of HEK293 cells transfected with the HA-IKKα and Flag-FKBP51 expression vectors, in the presence or not of FK506. Data are representative of two independent experiments. (**C**) Study of IκBα degradation induced by TNFα in A375 cells pre-incubated for 1 h in the absence or the presence of FK506, rapamycin and CSA, each compound at the concentration of 0.2 μg/ml.­ Data are representative of two independent experiments. (**D**) IκBα degradation induced by TNF-α in A375 cells pre-incubated for 1h in the absence or the presence of Rapamycin, CSA and the specific FKBP51 inhibitors SaFit1 and 2, each at the concentration of 0.2 μg/ml.­ Data are representative of two independent experiments. Both specific compounds inhibited TNF-α-induced IκBα degradation. (**E**) Measure by qPCR of CCND1 and FKBP1A levels showed that both SaFit1 and 2 significantly reduced TNF-α-induced of CCND1 levels of melanoma, in accordance with the NF-κB promoting ability of FKBP51. Modulation of cyclin D1 expression level was confirmed by immunoblot. Neither FKBP1A mRNA, nor FKBP12 protein, appeared to be modulated.

To define the mechanisms by which FKBP51 supported IKK kinase activity, we approached the study of FKBP51 interaction with IKK proteins. When expressed in HEK293T cells, FKBP51 bound to all three subunits of IKK, IKKα, IKKβ and IKKγ, with the binding to IKKα being most prominent (Figure 2a). The previously described FKBP51 interaction, with the IKK-activating kinase TAK1 (13), appears to be less pronounced (Figure 2a). It is feasible that the FKBP51/TAK1 association is IKK-mediated. Interestingly, FKBP51 knockdown reduced the binding of IKKγ to the catalytic IKK subunits, IKKα and IKKβ, under both transfection (Figure 2b) and endogenous (Figure 2c) conditions. These findings suggested a scaffolding role for FKBP51 in facilitating the assembly of IKK kinase complex. We next addressed whether the isomerase activity of FKBP51, in addition to its scaffolding function, of FKBP51 was also important for IKK regulation. For these studies, we incubated cells with FK506, a potent ligand of FKBP51 that inhibits its PPIase activity. FK506, indeed, reduced TNFα-stimulated IKK activity in A375 melanoma cells (Figure 3a). A moderate effect of FK506 on IKK activation was also detected in the FKBP51-knockdown cells, probably due to the incomplete depletion of FKBP51 in these cells or the action of other FK506-sensitive immunophilins. FK506 did not affect the physical interaction of FKBP51 with IKKα (Figure 3b), suggesting another mechanism of action. In accordance with the finding that FK506 impaired IKK function, FK506 attenuated TNFα-induced IκBα degradation (Figure 3c). A similar result was obtained with another ligand of FKBP51, rapamycin, but not with the cyclophilin A ligand cyclosporin. This result excluded a role for calcineurin phosphatase in the IKK-inhibitory action of FK506. The effect of FKBP51 inhibition on TNFα-induced IκBα degradation was confirmed using two compounds selective inhibitors of FKBP51, recently identified (22) namely SaFit1 and 2 (Figure 3d). Both compounds inhibited expression of cyclin D1, a transcriptional target of NF-κB (Figure 3e). The expression levels of FKBP12 did not appear to be modulated in a similar fashion, which ruled out an effect of SaFits on general transcription. The observation that rapamycin is more effective than the two compounds in reducing cyclin D1 transcript levels may depend on rapamycin capacity to inhibit other FKBPs involved in the NF-κB signaling (23). However, our result is also consistent with the notion that rapamycin has effects on Cyclin D1 expression independent of NF-κB (24).

### FKBP51 binds to TRAF2 and allows recruitment of IKK to TRAF2

**Figure 4.**
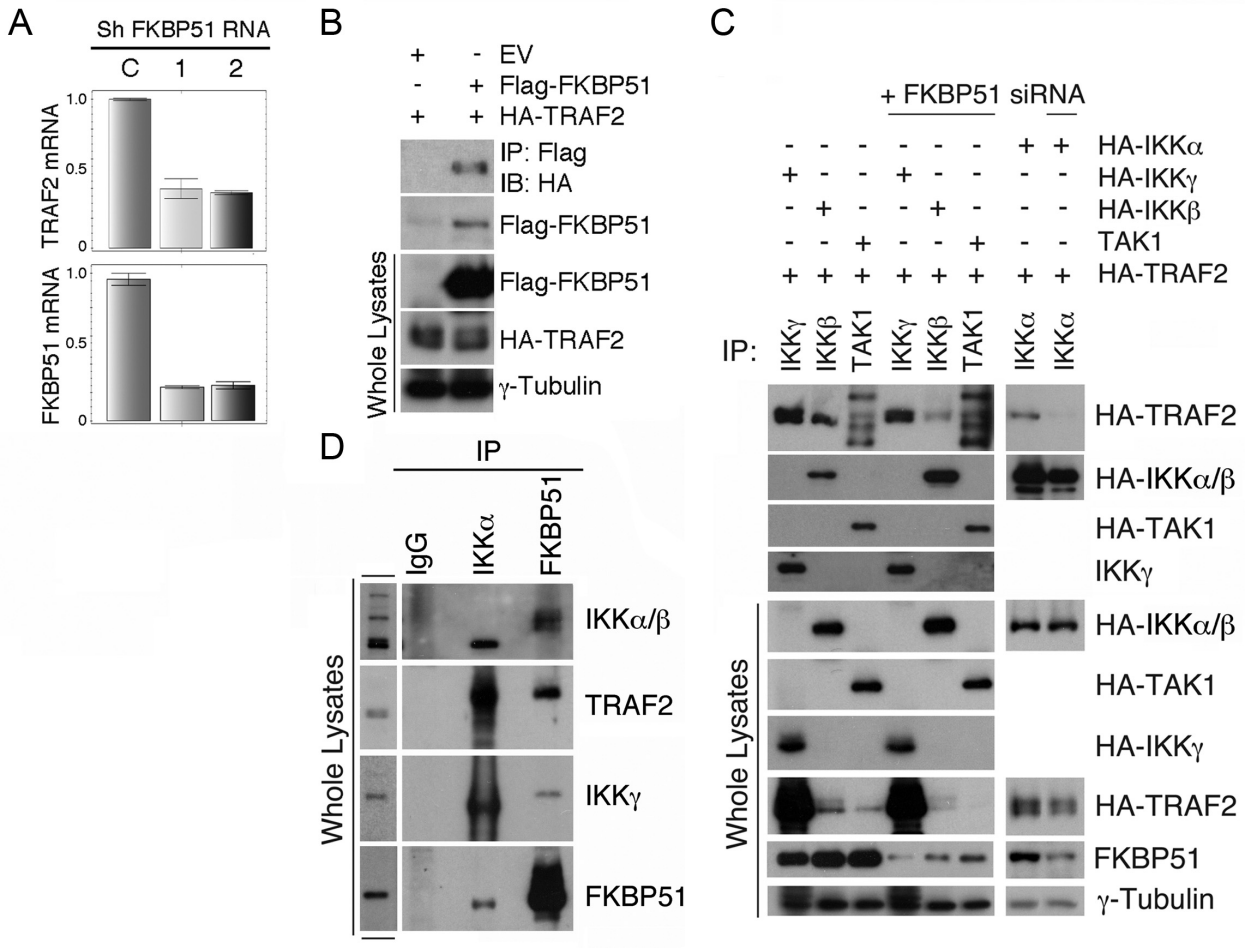
FKBP51 regulates interaction between TRAF2 and IKK. (**A**) Reduced TRAF2 levels in FKBP51 knocked down melanoma cells. QPCR analysis of the TRAF2 and FKBP51 mRNA levels in FKBP51-knocked down A375 cells. Data are representative of three independent experiments. (**B**) FKBP51 interacts with TRAF2. Immunoassay of HEK293 cells transfected with the HA-TRAF2 and Flag-FKBP51 expression vectors. Data are representative of three independent experiments. (**C**) FKBP51 silencing prevents TRAF2 interaction with IKK subunits. Immunoassay of whole cell extracts and immunoprecipitated proteins obtained by HEK293, silenced or not for FKBP51, and transfected with various combinations of HA-IKKα, -IKKβ, -IKKγ, -TAK1, -TRAF2 and Flag-FKBP51 expression vectors. Data are representative of four independent experiments. (**D**) FKBP51, IKK subunits and TRAF2 interact each other. Endogenous IKKα and FKBP51 were immunoprecipitated from whole lysates of A375 melanoma cells. Immunoprecipitated protein was then assayed in IB. Data are representative of two independent experiments.

TNF receptor-associated factor 2 (TRAF2) mediates TNFα-stimulated NF-κB signaling by catalyzing K63-linked polyubiquitination, a mechanism that facilitates the recruitment of IKK complex to its upstream kinase TAK1 (18–19). To examine the role of FKBP51 in regulating TRAF2 function, we first analyzed the effect of FKBP51 knockdown on the expression of the TRAF2 mRNA. Interestingly, the level of TRAF2 mRNA was reduced in FKBP51 knockdown melanoma cells (Figure 4a). The reduced TRAF2 mRNA expression in the FKBP51-knockdown cells might be due to the attenuated activity of NF-κB or yet to be identified factors. We over expressed TRAF2 in HEK293T cells, and found that TRAF2 physically interacted with FKBP51 (Figure 4b) as well as with TAK1 and each IKK components, α, β and γ (Figure 4c). FKBP51 silencing using siRNAs did not affect the TRAF2/TAK1 interaction, but reduced the binding of TRAF2 to IKK subunits, particularly IKKα and IKKβ (Figure 4c). Furthermore, we performed endogenous IKKα and FKBP51 co-IP assays in A375 melanoma cells. A representative result of 2 independent experiments is shown in Figure 4d. These results suggest that in addition to directly modulating IKK activity, FKBP51 may also promote the recruitment of IKK to TRAF2 and, thereby, facilitates IKK activation. The role of FKBP51 as a constitutive endogenous factor of IKK complexes is supported by the observation that this immunophilin is recovered from gel filtration profiles of melanoma cell lysates associated with IKK subunits (see supplementary information, Supplementary Figure S3). HSP90 was also eluted by gel filtration (Supplementary Figure S3), suggesting HSP90 can take part to FKBP51/IKK complex.

### The TPR domain of FKBP51 mediates IKK/TRAF2 interactions

**Figure 5.**
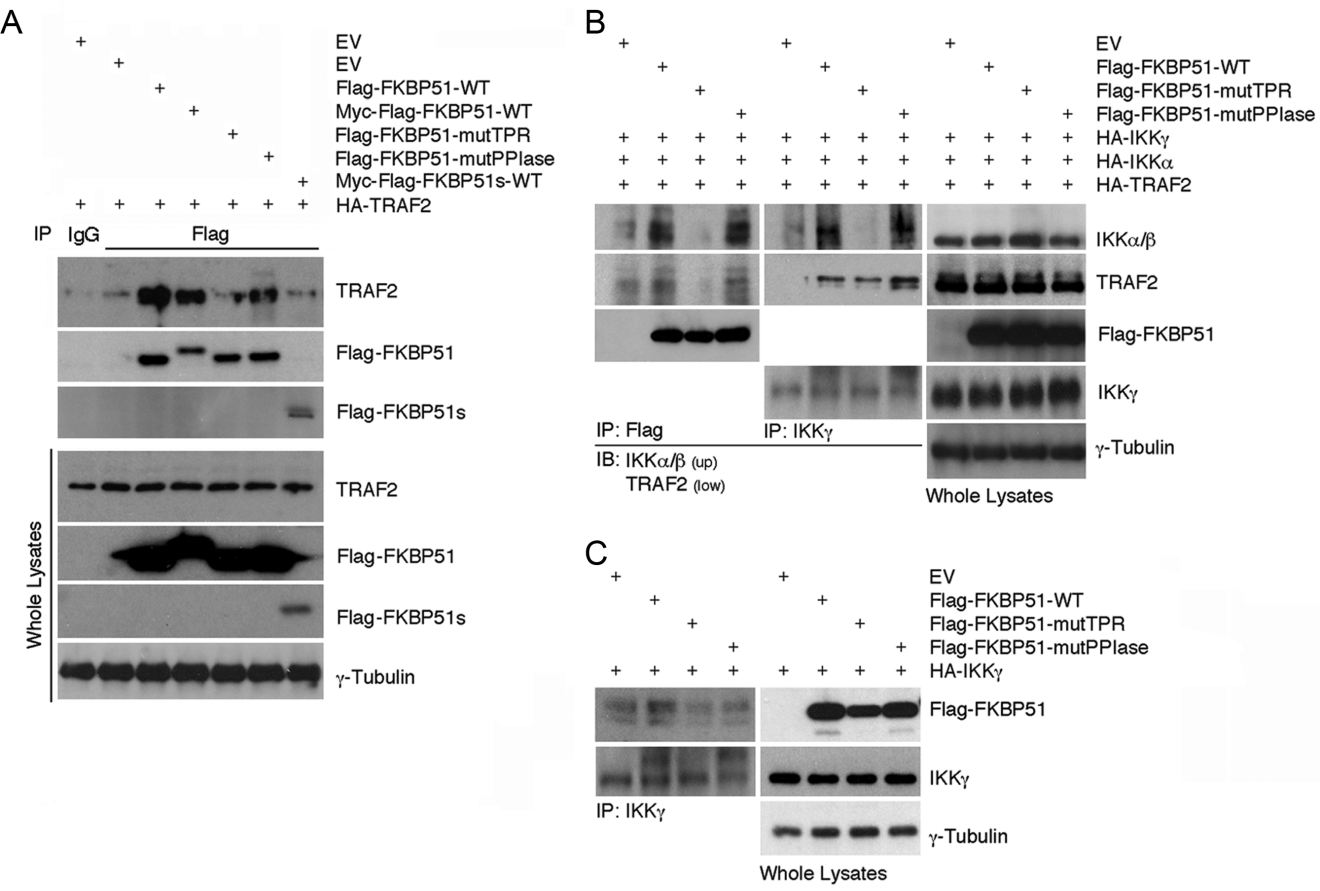
FKBP51 domains involved in binding to TRAF2 and IKK subunits. (**A**) Mutation of TPR domain impairs FKBP51/TRAF2 interaction. Immunoassay of HEK293 cells transfected with HA-TRAF2, Flag-FKBP51-mutTPR (carrying point mutation of TPR), FKBP51-mutPPIase (carrying point mutation of PPIase), Myc-Flag-FKBP51s (a truncated FKBP51 isoform lacking of TPR domains). Lysates were subjected to immunoprecipitation with anti-Flag and IB analysis was performed with anti-TRAF2 and anti-Flag (upper). IB of total lysates is also shown (lower). (**B**) TPR domain of FKBP51 promoted the association between the IKK subunits. Immunoassay of HEK293 cells transfected with the HA-TRAF2, -IKKα, -IKKγ and Flag-FKBP51-mutants expression vectors. Lysates were subjected to immunoprecipitation with anti-Flag, or anti-IKKγ and analyzed by IB with anti-IKKα/β and anti-TRAF2. IB of whole lysates is also shown. (**C**) Both TPR and PPIase domains of FKBP51 are involved in the IKKγ/FKBP51 interaction. Immunoassay of HEK293 cells transfected with HA-IKKγ and differents Flag-FKBP51 mutants. Lysates were subjected to immunoprecipitation with anti-IKKγ and analyzed by IB with anti-Flag. IB analysis of whole lysates is also shown. Data are representative of three independent experiments.

**Figure 6.**
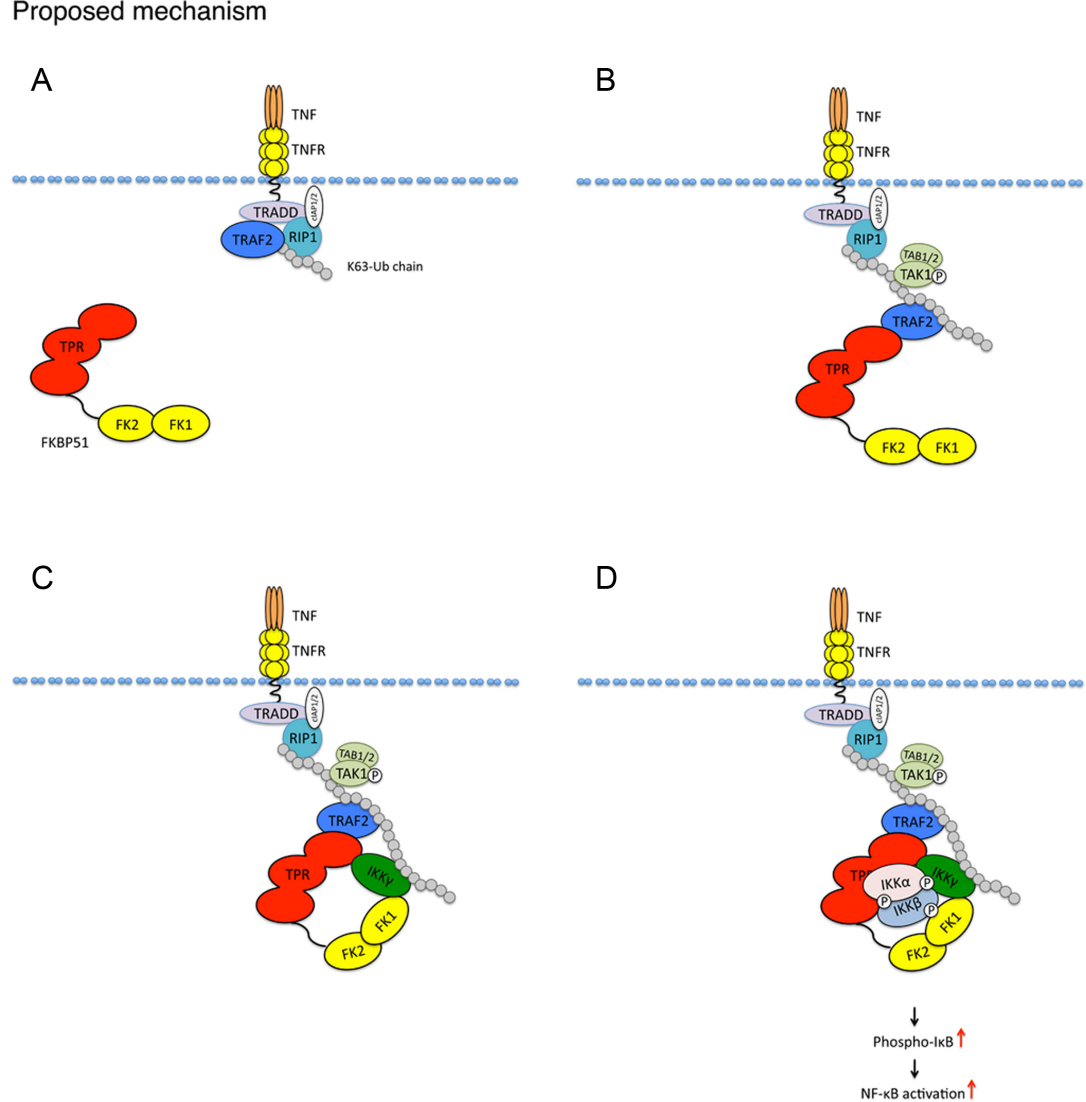
Proposed mechanism for the interaction of FKBP51 with NF-κB signaling proteins. (**A**) TNFα-binding to its receptor determines formation of RIP-induced K-63 ubiquitin chain. (**B**) TRAF2, promotes elongation of this non degradative ubiquitin chain and recruits TAK1 kinase complex. (**C**) TRAF2 interacts with TPR domain of FKBP51. IKKγ interacts with both FK and TPR domains of FKBP51. IKKγ and TRAF2 are also connected through K63 ubiquitin chain. (**D**) IKKα and β interact each others and with IKKγ and TRAF2 through the TPR domain.

To delineate functional domains of FKBP51 involved in modulation of the IKK/TRAF2 signaling complex, we employed FKBP51 mutants harboring disrupted PPIase (FKBP51^FD67DV^-Flag-FKBP51-mutPPIase) or TPR (FKBP51^K352A/R356A^-Flag-FKBP51-mutTPR) domain. Interestingly, the TPR mutant failed to bind TRAF2, whereas the PPIase mutant retained the TRAF2-binding function (Figure 5a). Consistent with this finding, a truncated form of FKBP51 (Myc-Flag-FKBP51s), lacking the TPR tandem repeats, barely interacted with TRAF2, suggesting the involvement of the TPR domain in FKBP51/TRAF2 interaction. The mutations in the TPR domain, but not the PPIase domain, of FKBP51 also abolished its ability to promote the association between IKKα/β and IKKγ (Figure 5b). In contrast, neither the TPR, nor the PPIase domain, was required for inducing the interaction between IKKγ and TRAF2 (Figure 5b). On the other hand, both TPR and PPIase domains of FKBP51 were important for the IKKγ/FKBP51 interaction, since this interaction has not been maintained by the TPR and PPIase mutants (Figure 5c). These findings also suggest that the TPR and PPIase domains of FKBP51 are not crucial for TRAF2/IKKγ binding, but the TPR domain is important for facilitating the IKK complex assembly and the recruitment of IKK to TRAF2. We hypothesize that FKBP51 binding to TRAF2 (through TPR) allows recruitment of IKKγ to TRAF2 (involving TPR and PPIase). Such TPR-mediated FKBP51 binding to TRAF2 does not appear to require HSP90 (see supplementary information, Supplementary Figure S4). It is possible that IKKγ isomerization may virtually initiate IKK complex assembly and autophosphorylation. A proposed mechanism of interaction is depicted in Figure 6. TRAF2 interacts with the TPR domain of FKBP51, whereas IKKγ interacts with both FK and TPR domains of FKBP51. The TPR domain promotes the assembly of IKK complex and allows IKK recruitment to TRAF2.

## DISCUSSION

Deregulation of the NF-κB regulatory pathway in cancer plays a major role in mediating chemo- and radio-resistance and cancer progression. In melanoma, a relevant role for aberrant NF-κB activation has been assigned to FKBP51 (7,8,11). Our present work confirms previous studies (7,8,11) showing a relevant role of FKBP51 in apoptosis resistance of melanoma and shed lights on the mechanisms by which this protein controls the cellular machinery that leads to activation of this transcription factor. Our study shows that FKBP51 interacts with all three subunits of IKK and contributes to mantaining the IKK complex formation and catalytic ability, at least in melanoma cells. The FKBP51 isomerase inhibitor FK506 did not prevent the IKK-FKBP51 interaction, but still hampered the kinase activity of IKK. In accordance with this finding, FK506 prevented TNFα-induced IκBα degradation. These results suggested that the PPIase activity of FKBP51 is important for supporting the enzymatic function of IKK. In further support of this hypothesis, we found that another FKBP inhibitor, rapamycin, but not the cyclophilin A inhibitor cyclosporin impaired TNFα-induced IκBα degradation. The fact that cyclosporin did not prevent TNFα-induced IκBα degradation ruled out a role for calcineurin inhibition in this effect.

By using two FKBP51 point mutants, with functionally compromised TPR or PPIase domains, and a truncated isoform lacking the TPR domain, we were able to determine the protein domains involved in interactions with IKK subunits. Additionally, we could verify that TRAF2 was a further interactor of FKBP51. According to our Co-IP experiments, both TPR and PPIase domains interacted with IKKγ, but only the TPR domain interacted with IKKα/β and with TRAF2. The latter finding is consistent with the insensitivity of the FKBP51-IKKα interaction to FK506 treatment. The strongest FKBP51 interaction was observed with IKKα. Moreover, the finding that FKBP51 knock down prevented interaction between IKKγ and IKKα, supported a role for this protein in IKK complex assembly. In addition to its scaffolding function, FKBP51 also seem to acts as an isomerase to regulate NF-κB. The isomerase inhibitor FK506 does not interfere with FKBP51/IKK binding or IKK complex assembly, but it nevertheless inhibits the kinase activity of IKK. Differently from Hinz *et al*. (25), that found FKBP51 and IKK not constitutively associated in HeLa, our results suggest a strict interaction between these proteins, consistent with the constitutive melanoma IKK activity (26).

We found that the TRAF2-IKKγ interaction is only in part affected by FKBP51 knock down. This is in accordance with the notion that the two proteins are also connected by a K63 ubiquitin chain; indeed in the canonical pathway, TRAF2 catalyze K63-linked polyubiquitination of receptor-interacting protein 1 (RIP1) in response to TNFα stimulation and TNFα receptor triggering; this noncanonical polyubiquitin chain recruits both TAK1 complex (consisting of TAK1, TAB2, and TAB3) and the IKK complex (consisting of IKKα, β, γ), by binding directly to the ubiquitin-binding domains present on TAB2 and IKKγ, respectively (27–29). In particular, TRAF2 seems to function as an E3 ubiquitin ligase that regulates the basal and inducible activation of NF-κB (30–33). It is possible that, in this step, TRAF2 binds to FKBP51, through TPR; this reinforces recruitment of IKKγ, which is still engaged by K63 ubiquitin chain, to TRAF2. FKBP51-TRAF2 interaction allows appropriate assembly of IKK complex subunits. Once hooked by polyubiquitinated RIP1, TAK1 is closer to the IKK complex and can phosphorylate it (26). FKBP51 promotes correct assembly of IKK subunits and modifies protein conformation for optimal enzimatic activity. The role of FKBP51 might not be restricted to TNFα-TRAF2-IKK signaling since FKBP51 was recently also identified as a TRAF3 and TRAF6 interactor (34).

Very recently, Erlejman *et al*. (23) found that another immunophilin namely FKBP52 promoted NF-κB activation in fibroblasts because it facilitated the nuclear translocation of the transcription factor. The authors also found that, in fibroblasts, FKBP51 counteracted the action of FKBP52 on NF-κB nuclear translocation. For this reason, the authors concluded that NF-κB activation was regulated in an antagonistic manner by the high molecular weight immunophilins FKBP51 and FKBP52, as it occured for steroid receptor. The reduced levels of NF-κB complexes in nuclei of FKBP51 knockdown cells, found in EMSA, accompanied by impairment of NF-κB-regulated gene expression underline the strict IKK dependence on FKBP51 and the prevalence of stimulatory effect in melanoma. Thus, even if FKBP51 regulated NF-κB at different levels of the activation signal, with effects that can be antagonistic, cell-context-related factors virtually affect the translocation rate of NF-κB heterocomplex, overcoming the suggested (23) inhibitory effects.

Taken together these results shed light on the mechanism by which FKBP51 allows NF-κB activation and suggest an essential role for this immunophilin in sustaining the constitutive activation of these transcriptional factors in melanoma. Finally, we show that the FKBP51 stimulating role on IKK signaling can be pharmacologically reduced, which results of this study may have an important translational implication in human therapy of this aggressive neoplasia.

## ACCESSION NUMBER

PDB ID: 4PY5.

## Supplementary Material

SUPPLEMENTARY DATA
