# Supplementary material for: FKBP51 employs both scaffold and isomerase functions to promote NF-κB activation in melanoma
Source: Nucleic Acids Res. 2015 Jun 22;43(14):6983–93. doi: 10.1093/nar/gkv615 (PMC4538817; doi:10.1093/nar/gkv615)
Supplement: SUPPLEMENTARY DATA [file supp_43_14_6983__index.html]

FKBP51 employs both scaffold and isomerase functions to promote NF-κB activation in melanoma — FKBP51 employs both scaffold and isomerase functions to promote NF-κB activation in melanoma — SUPPLEMENTARY DATA 

# FKBP51 employs both scaffold and isomerase functions to promote NF-κB activation in melanoma

## SUPPLEMENTARY DATA

- SUPPLEMENTARY DATA
