# Supplementary material for: FKBP51 employs both scaffold and isomerase functions to promote NF-κB activation in melanoma
Source: Nucleic Acids Res. 2015 Jun 22;43(14):6983–93. doi: 10.1093/nar/gkv615 (PMC4538817; doi:10.1093/nar/gkv615)
Supplement: SUPPLEMENTARY DATA [file supp_gkv615_nar-00899-x-2015-File008.pdf]

SUPPLEMENTARY INFORMATIONS

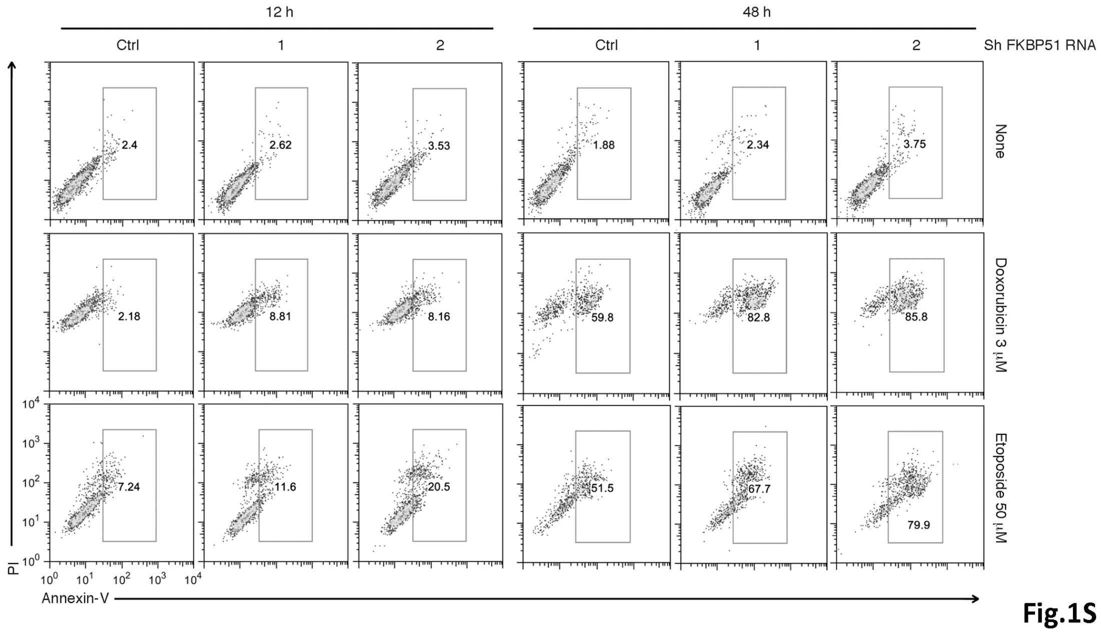

Fig.1S

Figure 1S. *Effect of FKBP51 knock down on melanoma apoptosis induced by doxorubicin and etoposide.* Flow cytometric histograms of annex-V/PI staining of two different FKBP51-knocked down A375 cell lines (1,2), stimulated with 3μM doxorubicin and 50μM etoposide. Cell was harvested after a 12h and 48h incubation.

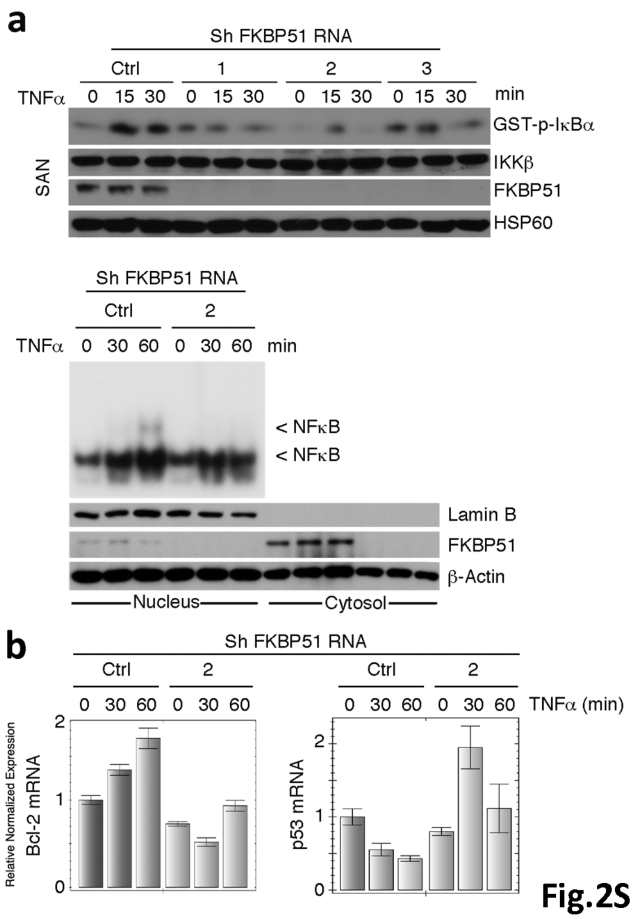

Fig.2S

Figure 2S. *Effect of FKBP51 knock down on NF-κB activation in SAN melanoma cell line.* (**a**, upper panel) KA of FKBP51-knocked-down SAN cells, stimulated with TNF-α. IKK activation was measured using GST-IκBα as substrate; IB assays showed the expression of IKKβ, FKBP51. HSP60 was used as loading control. (**a**, lower panel) EMSA of nuclear extracts of FKBP51-knocked-down SAN cells, stimulated with TNF-α. Binding of a ^{32}P-radiolabeled probe to NF-κB was visualized by autoradiography. IB shows the expression of FKBP51 in cell extracts and loading controls for cytoplasmic and nuclear extracts. (**b**) QPCR of the mRNA levels of the NF-κB transcriptional targets Bcl-2 and p53. Total RNA was isolated from cells as described in the materials and methods section, using co-amplified ribosomal β-Actin as an internal control for normalization. Oligo sequences are reported: hBCL-2-Fw: 5’-CTGCACCTGACGCCCTTCACC-3’ - hBCL-2-Rev: 5’-CACATGACCCCACCGAACTCAAAGA-3’ - hP53-Fw: 5’-ATCCTCACCATCATCACACTGG-3’ - hP53-Rev: 5’-TCTTGCGGAGATTCTCTTCCTC-3’. Data are representative of three independent experiments.

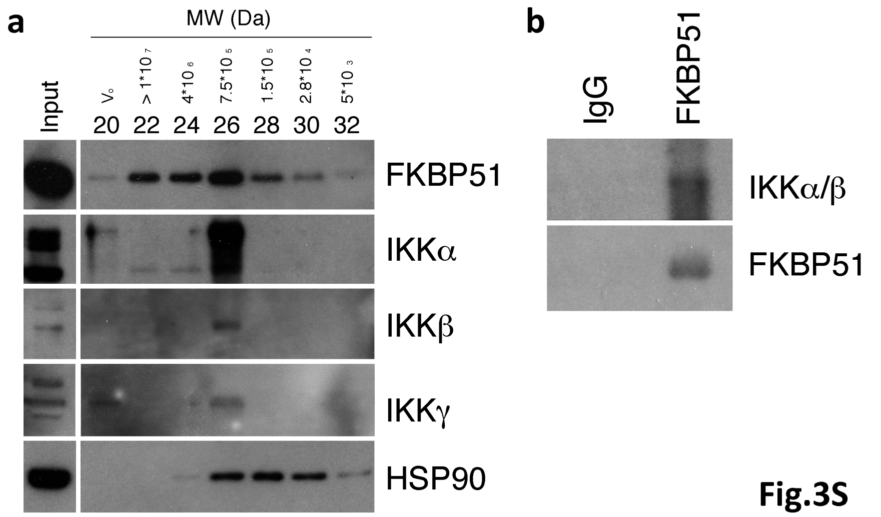

Figure 3S. *FKBP51 and IKK complex are constitutively associated.* (**a**) Melanoma A375 cells extracts were prepared and applied to a Superose 6 HR 30/30 gel filtration column, as reported by Hinz M et al. (JBC 2007). Fractions were collected and analyzed by Western blotting. Migration of marker proteins is indicated on the top of the figure; V0 is the dead volume of the column. (**b**) Immunoblot of immunoprecipitated FKBP51 in pooled fraction samples. FKBP51, recovered from gel filtration of melanoma cell lysates, coimmunoprecipitated with IKK subunits.

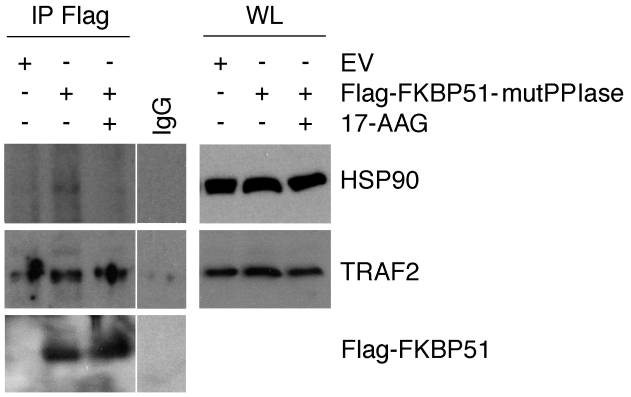

Fig.4S

Figure 4S. *17-AAG prevents HSP90 binding to FKBP51 but not FKBP51/TRAF2 interaction.* Immunoassay of HEK293 cells pretreated, or not, with 1 μM 17-(Allylamino)-17-demethoxygeldanamycin (17-AAG; Sigma). TPR-dependent binding of FKBP51 to TRAF2 was assayed using cells transfected with Flag-FKBP51-mutPPIase. Anti-flag (or anti mouse IgG) immunoprecipitated proteins were IB assayed with anti-HSP90 and TRAF2. IB analysis of total lysates is also shown (right panel). Binding of TRAF2 to FKBP51 is not prevented by 17-AAG that removes HSP90 from FKBP51 complex.
